# Highlights from the 56th Bürgenstock Conference on Stereochemistry 2023

**DOI:** 10.1039/d3sc90151c

**Published:** 2023-08-25

**Authors:** Marc Reid, Christopher J. Teskey

**Affiliations:** a WestCHEM Department of Pure & Applied Chemistry, University of Strathclyde Glasgow UK marc.reid.100@strath.ac.uk; b Institute of Organic Chemistry, RWTH Aachen University Landoltweg 1 52074 Aachen Germany christopher.teskey@rwth-aachen.de

## Abstract

Herein, we share an overview of the scientific highlights from speakers at the latest edition of the longstanding Bürgenstock Conference.

## Abstract 2

We share an overview of the scientific highlights from speakers at the latest edition of the longstanding Bürgenstock Conference.

## Graphical abstract

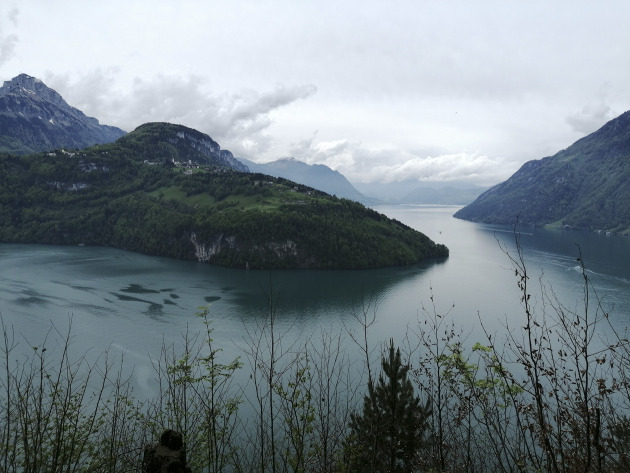

## 1 Introduction

Since the days of early pioneers including Dunitz, Barton, and Cornforth, the south of Switzerland has played a sustained role in hosting innumerable important conversations on the development of organic chemistry in the form of the Bürgenstock Conference. The 56th edition of this enduring and evolving conference kicked off on 7th May this year with thunder reverberating around the picturesque Swiss town of Brunnen. In the now regular setting of the Seehotel Waldstätterhof hotel, this meeting brings together chemists from many different backgrounds, in and beyond organic chemistry, to discuss challenges at the cutting edge of chemistry research. With a programme and participant list that remains secret until arrival, the only glimpses of who and what to expect come from the people you meet on the way from the train station to the hotel. Excitement builds with every step!

With 135 participants this year, the meeting was one of the largest in its illustrious history, and took place under the stewardship of 2023 President, Prof. Alois Fürstner (Max-Planck-Institut für Kohlenforschung) who welcomed everyone over the opening drinks reception. It was noted that the weather, traditionally (perhaps allegedly) the responsibility of the Vice-President – this year Prof. Erick Carreira (ETH Zürich) – had some room for improvement. Prof. Fürstner also introduced the 17 JSP Early Career Fellows – 14 from academia, 3 from industry – competitively supported to attend and participate in the conference. The research topics represented by the fellows spanned depolymerization strategies,^1^ heterobimetallics,^2^ photocatalysis,^3^ reactive intermediates,^4^ ligand design,^5^ heterocycle synthesis,^6^ computer vision-enabled reaction monitoring,^7^ cobalt catalysis,^8^ and cyclopropane synthesis.^9^ As a final part of the introductions, the guest of honour for this year, Prof. Andreas Pfaltz (Emeritus; Universität Basel), was announced. In a stroke of coincidence, the honoured Pfaltz, a pioneer in the field of asymmetric hydrogenation,^10^ allowed everyone to share in his birthday celebrations a few days after the conference began.

## 2 Programming and playing with conformation

The scientific programme began after dinner on Sunday evening with a masterclass in topology from Prof. Tanja Gaich (University of Konstanz) who presented the total synthesis from her group of the unusual complex diterpene, canataxpropellane.^11,12^ The successful route involved cascade Diels–Alder reactions followed by a photochemical [2 + 2] reaction to construct the core in an efficient manner. After first showing us the successful route, Prof. Gaich dove into the details of unsuccessful paths along the journey. Of central importance to solving this challenging natural product synthesis was understanding the structures by analysing the conformations from many different perspectives. Indeed, it was made clear to the audience how vitally important it was to consider abrupt changes in conformation with each transformation, as the core skeleton evolved towards the targeted end product.

The theme of conformation was continued by Prof. Ryan Gilmour (University of Münster) the following morning when he shared his group’s wide-ranging contributions to contra-thermodynamic alkene isomerisations,^13^ in a talk that balanced contemporary synthesis with historical insights. Taking inspiration from nature, the Gilmour group has leveraged selective photoisomerisation of many classes of alkenes based on the selective photoabsorption of the thermodynamically stable geometrical isomer. Among recent contributions, the group’s strategy was showcased in the stereochemically programmable construction of polyenes, including 13-*cis*-retinoic acid (isotretinoin) and 9-*cis*-retinoic acid (alitretinoin).

## 3 Hydrides, differently

Prof. Shunsuke Chiba (Nanyang Technological University) was the next speaker, sharing how his group has built a research programme around unexpected reactivity of sodium hydride. Contrary to the commonly known application of NaH as a base rather than a reducing agent, Prof. Chiba demonstrated that, in combination with metal halides, metal hydrides can be employed as uniquely selective reductants for a range of functional groups. In one of many intriguing case studies shared, the Bürgenstock audience learned of the controllable ability to selectively reduce carboxamides to either primary alcohols or dimethylamines, through the choice of zinc salt partnered with sodium hydride.^14^

## 4 The world within us

Delivering a fascinating conceptual pivot, Prof. Emily Balskus (Harvard University) came to the stage to share an overview of her team’s work to decode the human gut microbiome using chemistry. She explained that only a small fraction of genes in the microbial environment are understood and the human colon is one of the most complex of these environments. One of the interests of her team is colibactin, a small molecule genotoxin that, as a DNA cross-linking agent, is implicated in colorectal cancer. By studying uncharacterised biosynthetic enzymes encoded in the *clb* gene cluster before isolation, Balskus and her team have pioneered a new approach for structural elucidation and investigation of the biological role of this unstable natural product.^15^

## 5 Talks for all, by all

In a rare and defining characteristic of the Bürgenstock Conference, the poster session welcomed (and encouraged) participation from both new and well-established researchers. This served as one of two such sessions held during the conference. As a taster for the pre-dinner discussions, six junior participants in the conference were invited to give flash talks to share progress in the fields they are helping pioneer. The Swiss-standard time-keeping meant that the end of each talk was strictly marked by the distinctive ‘mooing’, politely indicating that further discussions should continue during the poster session. As a microcosm of the plenary sessions, these talks introduced the audience to a broad range of endeavours, including machine learning-assisted enzyme engineering,^16^ natural product synthesis,^17^ energy-harvesting materials design,^18^ bio-orthogonal synthetic methodology,^19^ new platforms for high throughput drug discovery,^20^ and new methods in organic electrochemistry.^21^

## 6 Cell division from the bottom up

The vibrant poster session took attendees into nightfall and to the final talk of the day, delivered by Prof. Petra Schwille (Max Planck Institute for Biochemistry). In a talk that challenged the audience to consider the origins of life from the perspective of how cells are made, more so than how they replicate, Schwille spoke about the historical and philosophical discussion of how to define life. Through this unique lens, the audience appreciated how a physicist doing biology can lead to innovations in chemistry. The case in point was Prof. Schwille’s retelling of the story towards her team’s pioneering *in vitro* realisation of the minimal chemical machinery needed to affect cell contraction and thus division.^22^

## 7 Metalloenzymes understanding, inside out

Prof. Serena DeBeer (Max Planck Institute for Chemical Energy Conversion) turned attention towards analytical instrumentation, sharing her work on deciphering nitrogenase structures with X-ray emission spectroscopy (XES). Following photoionisation to eject an electron from the core 1s orbital of the metal centre, XES enables observation of the resulting fluorescent transition of a ligand-centred valence electron to the 1s hole. In a story that sparked jovial support from the audience used to sounding alarms whenever a typographically erroneously 5-bonded carbon is spotted in skeletal drawings, Prof. DeBeer exemplified how XES had been applied in her team to elucidate the highly unusual 6-coordinate carbon in a Fe_6_C core of nitrogenase cofactors.^23^

## 8 Meteoric transformations to understanding life

Next, Prof. Joseph Moran (University of Strasbourg) spoke about his group’s approach to understanding prebiotic chemistry. He showed clear evidence that most of the molecules on the Krebs cycle can be formed under relatively mild conditions, in transformations often mediated by transition metal salts. In an investigation of mechanisms by which hydrogen (as a geologically-relevant reductant) could mediate the reverse Krebs cycle, Moran’s team demonstrated that sources of meteorite powder could catalyse the hydrogenation of fumerate to succinate, and oxaloacetate to malate.^24^

## 9 From iridium hydrides to nitrenoids

Wednesday began with a lecture from Prof. Tim Donohoe (University of Oxford) who shared the journey his group had taken to expand the utility of interrupted hydrogen borrowing catalysis to deliver a suite of powerful alkylation strategies. Among the highlights, Prof. Donohoe exemplified the versatility of the Ph* (1,2,3,4,5-pentamethylphenyl-) ketone substituent as a masked carboxylic acid. Under iridium-catalyzed conditions, Donohoe’s team have shown that Ph* methyl ketone can serve as an alkylating reagent in hydrogen borrowing strategies, with Ph* then cleaved under aqueous acidic conditions to reveal the carboxylic acid.^25^

After the coffee break, Prof. Sukbok Chang (Korea Advanced Institute of Science and Technology, KAIST) presented the many discoveries that his group have made leveraging nitrene intermediates for catalytic C–H amination strategies. These reports included a range of innovations in the use of iridium to stabilise nitrenes revealed from the programmable, decarboxylative degradation of dioxazolones.^26^ From among the Chang group’s most recent pursuits at the time of the conference, the audience heard of emerging detailed studies into the mechanism of acyl nitrenoid formation with rhodium, using photocrystallography to capture fleeting intermediates.

## 10 Process chemistry takes centre stage

Balancing a range of high-quality research discussions from an academic perspective, Dr Margaret Faul (Amgen) gave an industry-focussed perspective on process development, from discovery to scale-up and the time pressures affecting the choice of process analytics. After an overview of what Amgen stand for, Dr Faul shared on the syntheses of Tapotoclax, Murizatoclax, Simeprevir, and refinement of the process to manufacture Kyprolis. In one particular process chemistry masterclass, Dr Faul shared the story of candidate AMG 176 from the company’s oncology pipeline, made using a key ring closing metathesis (RCM) step to furnish a 16-membered macrocycle.^27^ Far from trivial, this case study exemplified the combined value of high throughput experimentation, computational chemistry, and reaction kinetics not only to understand the selectivity for the desired step *versus* dimerisation side products, but how this knowledge could be applied to process-relevant parameters such as *E*-factor (kg of waste per kg of useful product). This industry-focused session was complemented by research endeavours of industrial JSP fellows, whose work covered innovations in scalable pyrazole synthesis,^28^ synthesis with spirocycles,^29^ and machine-learning enabled reaction optimisation.^30^

The same afternoon saw a return to a rapid relay of short talks, further expanding the conference coverage to areas including innovations in iron catalysis,^31^ Lewis base catalysis,^32^ and literature-based synthetic yield predictions in synthesis.^33^

## 11 Back to natural products

The penultimate day of the Bürgenstock Conference ended with a lecture by Prof. Ryan Shenvi (Scripps) who recharged the audience’s post-dinner attention with some opening slides on chess problems. He went on to draw an analogy to the complexity and variety in chemistry, expanding on how his research group have been using total synthesis as a tool to navigate chemical space. Highlights included the total synthesis of GB18 on gram scale and the pharmacological properties of both the natural product and analogues were explored.^34^ Prof. Shenvi ended by posing the open-ended question of why it is we are focussed on natural products rather than their analogues. A further natural product family, the salvinorins had also piqued the interest of the Shenvi group as a possible solution to the opioid crisis, and Prof. Shenvi elegantly took us through their investigations in this area to conclude the day’s lectures.

## 12 Golden hour

The final day began with a talk by Prof. Didier Bourissou who took the audience back to the realms of transition metal catalysis. Specifically, Bourissou explained how his team has pioneered Au(i)/Au(iii) catalytic methods. Not only have they exploited the excellent Lewis acidic properties but considered judiciously-selected P,N ligand structures (*e.g.* MeDalPhos) that facilitate oxidative addition at Au(i). Excitingly, his group have applied this catalytic modality to combined aryl iodide oxidative addition and π-activation of alkenols to deliver a library of tetrahydrofurans, pyrans, and oxepanes.^35^

## 13 Deeper computation at larger scales

The honour of the final talk of the conference was given to one of the President’s colleagues from the Max-Planck-Institut für Kohlenforschung in Mülheim. Prof. Frank Neese gave a wide-ranging overview of his research and commercial ventures in computational chemistry. Neese shared some recent results on a very unusual triplet bismuthinidene as well as presenting the most recent updates to his widely-used ORCA quantum chemistry program system.^36^ A fantastic scientific talk, interspersed with philosophy left us with a final thought best summarised by the quote from Max Planck: “*Experiment is the only means of knowledge at our disposal. Everything else is poetry, imagination*.”

## 14 Final remarks

The broader experience of this, one of chemistry’s most enduring conferences, included a recreational afternoon to explore the scenic offerings of Brunnen (on a day that the rain relented), and rich conversations with a diversity of fascinating and fascinated scientific minds. The conference President treated the audience in his care to a classical concert, with delicious meals helping bookend the week-long chemistry tour de force.

The Bürgenstock Conference has appreciably expanded in its scientific remit since its inception in 1965. While maintaining a foothold in stereochemistry through links to the modern trailblazing work in natural product synthesis, the conference is, in these authors’ shared experience, all the more valuable for representing the very latest developments in synthesis, catalysis, chemical biology, computational chemistry, and so much more. Thankfully, with plans for the 57th conference now in motion, this cornerstone of chemistry research shows no signs of ever slowing down. We dare speak for all the conference attendees in saying we are grateful to have been a part of it.

## Author contributions

Both authors contributed to the drafting of this conference report.

## Conflicts of interest

The authors declare no conflicts of interest.

## Supplementary Material
